# Transient expression of CCL21as recombinant protein in tomato

**DOI:** 10.1016/j.btre.2017.11.007

**Published:** 2017-11-26

**Authors:** Maria Beihaghi, Hasan Marashi, Abdolreza Bagheri, Mojtaba Sankian

**Affiliations:** a College of Agriculture, Ferdowsi University of Mashhad, Mashhad, Iran; b BuAli Research Institute, Mashhad University of Medical Sciences, Mashhad, Iran

**Keywords:** Recombinant vaccine, Agroinfiltration, Tomato, Transient gene expression, Migration, Scratch assay

## Abstract

The main goal of this study was to investigate the possibility of expressing recombinant protein of C-C chemokine ligand 21 (CCL21) in *Solanum lycopersicum* via agroinfiltration. CCL21 is a chemokine can be used for anti-metastatic of cancer cell lines. To examine the expression of CCL21 protein in *S. lycopersicum*, the construct of *ccl21* was synthesized. This construct was cloned into pBI121 and the resulting CCL21 plasmid was agro-infiltrated into *S. lycopersicum* leaves. Within three days after infiltration, Expression of the foreign gene was confirmed by quantitative Real-time PCR. A recombinant CCL21 protein was immunogenically detected by western blot, dot blot and ELISA assay. And results showed that the foreign gene was expressed in the transformed leaves in high level. Also scratch assay was used to investigate the role of this protein in anti-metastatic function. The results demonstrated anti-metastatic of cancer cells in the presence of this protein.

## Highlights

• Applied use of plants as bioreactors for production of recombinant proteins.
• The present study is the first to report gene expression of ccl21 construct in tomato via agro infiltration to use this plant as oral vaccine.
• To investigate the role of this protein in anti-metastatic function on cancer cells

## 1 Introduction

Chemokines and their receptors play essential roles in leukocyte recruitment, tumor cell growth and metastasis [[1], [2]]. Among them, C—C chemokine ligand 21, Secondary lymphoid tissue chemokine (CCL21), exerts antitumor immunity by co-localizing dendritic cells and T cells at the tumor sites [[3], [2]]. Application of plants as bioreactors for production of recombinant proteins has emerged as a molecular farming over the past two decades. [3]. Many strategies have been proposed for the enhancement of recombinant protein expression including; use of strong promoters, chloroplast transformation [3], signal peptide codon optimization and untranslated leader sequences [4]. The Long time required for the generation of transformed plants expressing foreign antigens is another limitation for the production of recombinant proteins [5]. Transient gene expression methods are appropriate alternatives to stable transformation because they allow an inexpensive and rapid method for expression of foreign gene(s) in plant tissues. This method can be carried out in many ways including protoplast transformation, vacuum infiltration [6], agroinfiltration [7]. Among them, agroinfiltration takes advantages of a cost effective, simple and rapid procedure. In this method, the suspension of *Agrobacterium tumefactions* caring the gene(s) of interest is infiltrated into plant leaves using a needle-free syringe [7]. This technique has been carried out in a variety of plants [8] with different experimental purposes [9]. However, there have been few reports on production of recombinant proteins in plant systems via transient gene expression. The main reason is that transient gene expression assays are not as appropriate as stable transformation for production of recombinant vaccines [10]. However, recombinant protein produced via transient gene expression can be used for the production of specific antibodies, which can then be used in diagnosis and molecular detection. Moreover, transient expression assay can be carried out as a quick method to investigate efficiency of a candidate antigen for inducing immunogenic response in animal models [11]. In this study, we investigated the production of an immunogenic recombinant protein of CCL21 by agroinfiltration of tomato leaves. The gene construct is composed of a DNA fragment encoding 134 amino acids of CCL21 protein and part of Interleukin 1 beta (IL-1β). Also, Kozak sequence as a ribosome binding site and SEKDEL sequence as signal peptide were inserted in gene construct. Wound healing is a dynamic, and interactive process involving soluble mediators, blood cells, extracellular matrix, and mesenchymal cells. [4]. The aim of the present study was to investigate the expression of *ccl21* in tomato leaves via agroinfiltration and effect of CCl21 that expressed in tomato leaves on the proliferation of human cancer cell line and also their migration capacity in a cultured monolayer, serving as in vitro wound model.

## 2 Materials and methods

### 2.1 Construction of expression cassette

**Fig. 1:**
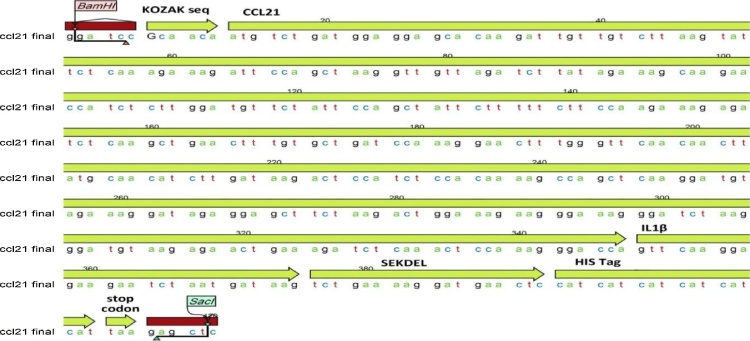
Schematic diagram of the plant expression system construct used for agro-infiltration.

DNA encoding 134 amino acids of CCL21 and part of IL-1β capsid protein was designed as the main part of expression cassette. A ribosome binding site, Kozak sequence GCCACC, was introduced prior to the start codon. This sequence has been reported to enhance translation efficiency [12]. An endoplasmic reticulum signal peptide called SEKDEL, which has been reported to increase recombinant protein accumulation in plant tissues [13], was attached to 3′ end just before stop codon. Start codon (AUG) and stop codon (UAA) were also added into the 5′ and 3′ ends of the construct, respectively. Recognition sites of *Bam*HI and *Sac*I restriction enzymes were introduced into the 5′ and 3′ ends of the synthetic gene, respectively. The resulted chimeric gene was optimized based on codon usage pattern of tomato taken from http://www.kazusa.or.jp/codon/. The optimized chimeric gene was cloned into the pGem-T Easy vector (Biorey, China) (Fig. 1).

### 2.2 Construction of binary vector

The synthetic *ccl21* gene was removed from pGem-TEasy vector by digestion with *Bam*HI and *Sac*I and was inserted into the binary vector pBI121, yielding pBI121-CCL21 vector (Fig. 1). The ligation reaction mixture was used to transform *E*. *coli* strain DH5-α and kanamycin-resistant colonies were isolated after overnight incubation at 37 °C. After amplification, the plasmid was extracted from bacterial cells using alkaline lysis method. The plasmid was introduced into *Agrobacterium tumefaciens* strain GV3101 by heat shock method. Transformed cells were screened by kanamycin-resistance and PCR.

### 2.3 Plant transformation via agroinfiltration

A single colony of *Agrobacterium* containing, pBI121-*ccl21* plasmid was cultured for 48 h in LB medium (NaCl 10 g/L, yeast extract 5 g/L, tryptone 5 g/L) supplemented with gentamicin 10 mg/L, rifampicin 50 mg/L and kanamycin 50 mg/L. After reaching a cell density of OD600 = 1.5, the culture was centrifuged, supernatant was discarded and the pellet was resuspended in infiltration medium (10 mM MgCl2, 10 mM MES pH 5.6, and 150 μM acetosyringone), adjusted to OD600 = 0.5. The suspension was then incubated for 2 h at room temperature before being transferred to tomato leaves with a needle-free syringe, as described by Sparkes et al. [14]. Tomato leaves were placed in growth chamber for three days under 25 °C before being analyzed.

### 2.4 R T- PCR assay

RT- PCR assay was performed to analyze gene expression at transcription level. Total RNA was extracted from infiltrated leaf tissue (parstus kit;Cat. No A101231) and complementary DNA (cDNA) was synthesized via reverse transcription using oligo(dT) primer (Parstous kit; Cat.No C101131). The resulting cDNA mixtures were used as templates for Real Time PCR. Expression of the synthetic gene was quantitatively analyzed using a Real-Time PCR system (BioRad). Real Time PCR was carried out in a 20 μL reaction volume containing 1 μM of each primer and 10 μL of SYBR Green Real time PCR master mix(Parstous Kit). Quantitative Real Time PCR experiments were performed in duplicate for each sample. Forward and reverse primers for Real Time PCR were 5′ GGGTTCAACAACTTATGC 3′ and 5′ CTTTCCCTTCTTTCCAGT 3′, respectively.

### 2.5 Protein dot blot assay and western blot assay

Production of recombinant CCL21 protein in transgenic leaves was evaluated by standard protein dot blot assay. So, 10 μL of protein samples from infiltrated leaves was dotted on the nitrocellulose membrane and the membrane allowed to get dried. The membrane was incubated with BSA as blocking solution for 1 h. After incubation, the membrane was washed three times with PBST/PBS and then, membrane was incubated with conjugated antibody His for 1 h in37 °C, and again washed three times with PBST/PBS and incubated with DAB (diaminobenzidine) substrate. A small volume of commercial CCl21 antigene that has histidine tag (about 5 μL) was used as positive control and the same volume of protein obtained from wild type plant was used as negative control. For western blot Sample proteins were resolved on reducing 12% SDS-PAGE and then visualized after Coomassie Brilliant Blue staining. For further characterization, the separated proteins on SDS-PAGE were transferred to PVDF membrane by electroblotting (Biometra) for 2 h room temperature at 120 V. The membrane was then blocked with blocking buffer [BSA in PBS] for 1 h at room temperature or overnight at 4 °C. The membrane was washed three times with washing buffer [PBST], each for 10 min at room temperature. The membrane was probed with the conjugated anti–6x His tag^®^ mouse monoclonal antibody (Sigma-Aldrich) for overnight at 4 °C or 1 h at room temperature and washed again three times with washing buffer. Finally the protein bounds were visualized by staining the membrane with DAB (Di-aminobenzidine) substrate.

### 2.6 Enzyme-linked immunosorbent assay (ELISA)

Expression of recombinant protein was further evaluated by ELISA assay. ELISA plate was coated with total soluble proteins from the wild type and the transformed plants and known CCL21 antigen at 37 °C for one hour; followed by incubation with 1% bovine serum albumin (BSA) in PBS for 2 h at 37 °C to prevent non-specific binding. The wells were washed by PBST/PBS and incubated with antiserum (Anti His tag) reactivated against CCL21 (1:1000 dilutions). Wells were developed with TMB substrate; the color reaction was stopped by 2N H2SO4 and read at 405 nm of wavelength.

### 2.7 In vitro wound assay

The scratch assay was performed on cells to study the effect of protein extraction of transformed leaves of tomato on cell migration. In vitro wounds were induced by modified protocol of described by [21]. MCF7 cells were seeded in 6-well plates at densities of 6 × 205 cells/well in the growth medium. After reaching near 90% confluency, the cells were switched to the basal media for 24 h. Then the cells, grown as a monolayer, were scratched using a sterile micropipetting tip. The tip was drawn firmly across the diameter of the dish. Cells were washed by PBS in order to remove the loosened debris. A range of concentrations (2 mg/ml) of plant extracts were added to a set of each wells for each dose. The cells were treated with equivalent amount of DMSO, as controls. For migration assay, cultures were rinsed twice with PBS, fixed by absolute methanol, stained by Giemsa, and examined by a light microscope, equipped with a calibrated ocular; at a magnification of 40x. Images were taken immediately after wounding followed by further imaging at 24, 48 and, 72 h time points. Cell migration rates were quantified by measuring the change in the wound area (pixels), using Image J software.

### 2.8 Statistical analysis

Each experiment was performed at least three times. Data were analyzed by one-way ANOVA. Also Duncan test was performed as post hoc analysis. Differences at the 95% level were considered to be significant. Normality, homogeneity of variances and data independency were evaluated before all the analyses. Results were expressed as mean ± S.D. using STATISTICA software

## 3 Results

### 3.1 Transient expression of ccl21 by real −time PCR assay

**Fig. 2:**
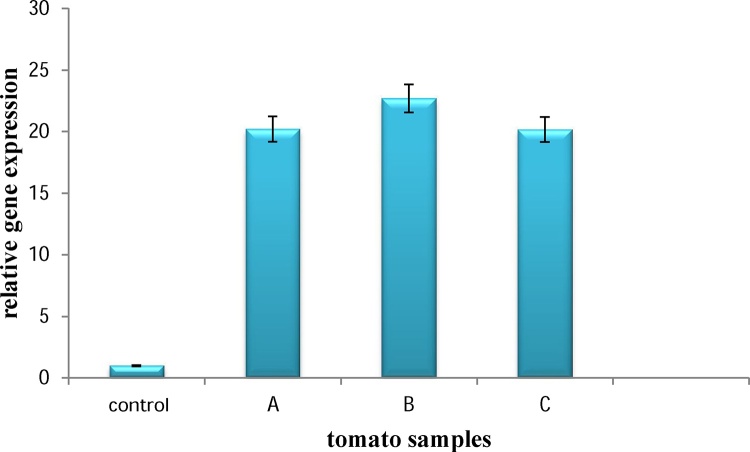
Quantitative measurement of *ccl21* gene transcription in transfected leaves of tomato via Real Time PCR. A, B and C: Three samples of transfected plants, Control: Negative control (non-transfected plant).

Expression of *ccl21* was evaluated at transcription level using quantitative Real-time PCR. Three samples of transformed leaves were used for Real-time PCR. Results showed that the foreign gene was transcribed in infiltrated leaves (Fig. 2).

### 3.2 Detection of recombinant CCL21 by standard protein dot blot and western blot assay

**Fig. 3:**
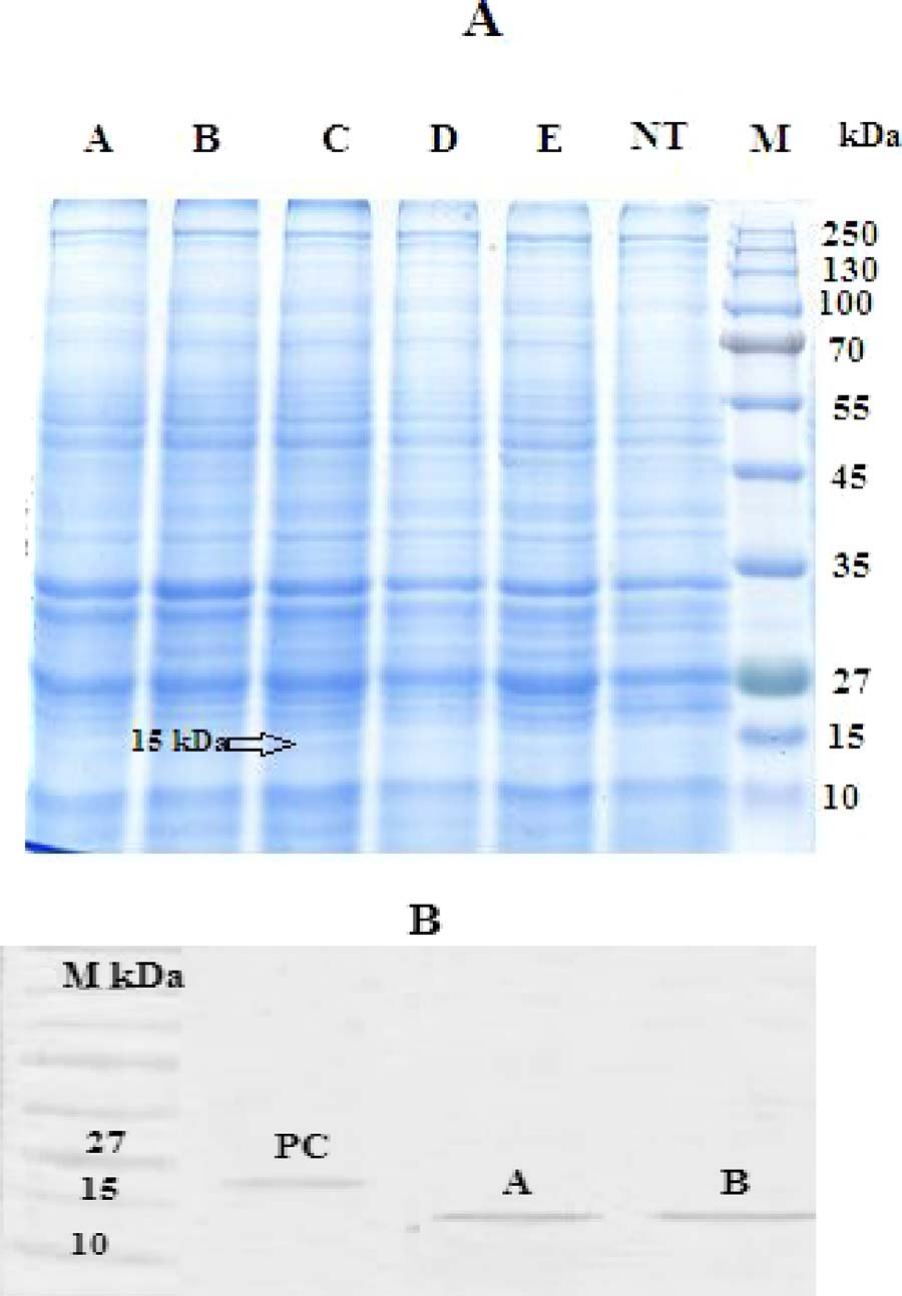
(A) SDS-PAGE analysis on transfected and non-transfected plants. lane A,B,C,D,F: transfected plants, lane NT: non-transfected plant (negative control). lane1 ladder: Fermentase Prestained Protein Ladder (SM1811). (B) Detection of CCL21 in protein extracts from the leaves of transfected tomato by western blot analysis using conjugated anti–6x His tag^®^ mouse monoclonal antibody (Sigma-Aldrich). M: Protein Marker (Fermentas), pc: commercial CCK21 antigen, NT: non-transfected plant, A,B: transfected plant.

**Fig. 4:**
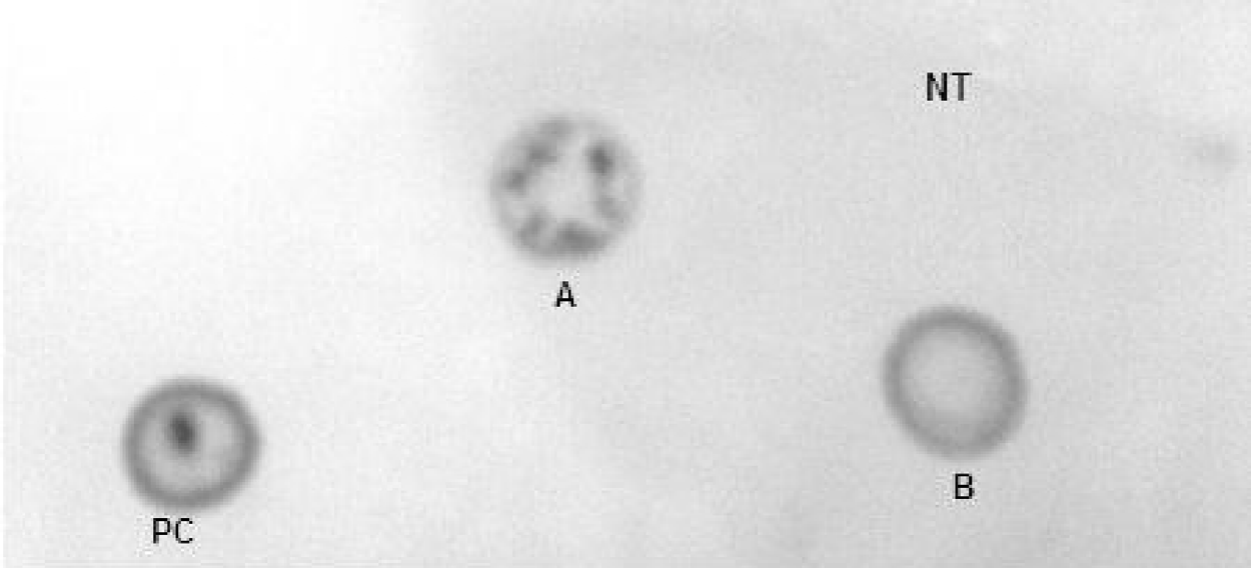
Protein dot blot for detection of recombinant protein in transfected leaves of tomato. (1): positive control, (2, 3): protein sample of transfected plants and (4): protein sample of non-transfected plant.

A protein with estimated 15.5 KD molecular weight was detected in western blot analysis using conjugated anti–6 x His tag^®^ mouse monoclonal antibody. No protein band was observed in protein samples of non-transfected plants as wild type plants (Fig. 3B). Dot blot results confirmed expression of the foreign gene at translation level, whereas no signal was observed for wild type plants (Fig. 4).

### 3.3 Quantitative expression of the recombinant protein by ELISA

**Fig. 5:**
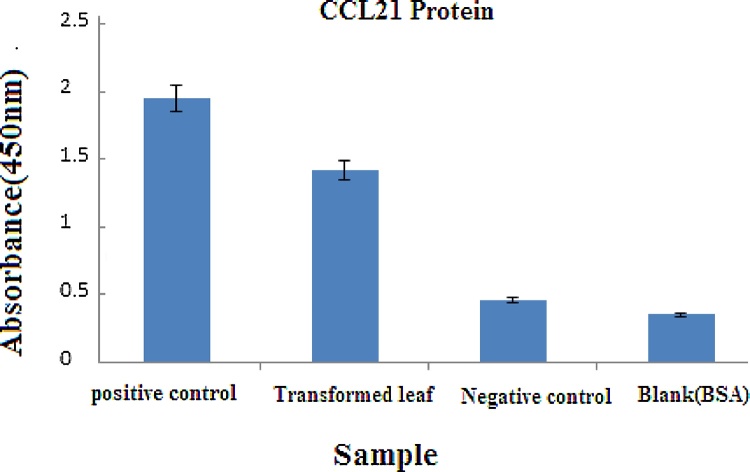
Quantification of recombinant CCL21 expression in transfected plants by ELISA.

Expression of the recombinant protein was further quantitatively measured by ELISA (Fig. 5). Production of the recombinant protein was quite high in transfected leaves. In contrast, no strong signal was observed for non-transfected plants. ELISA analysis of transiently expressed CCL21 from the total protein extraction of infiltrated leaves confirmed the expression of CCL21 that was specifically recognized by conjugated anti-His antibody.

### 3.4 Migration of cancer cell line into in vitro wounds

**Fig. 6:**
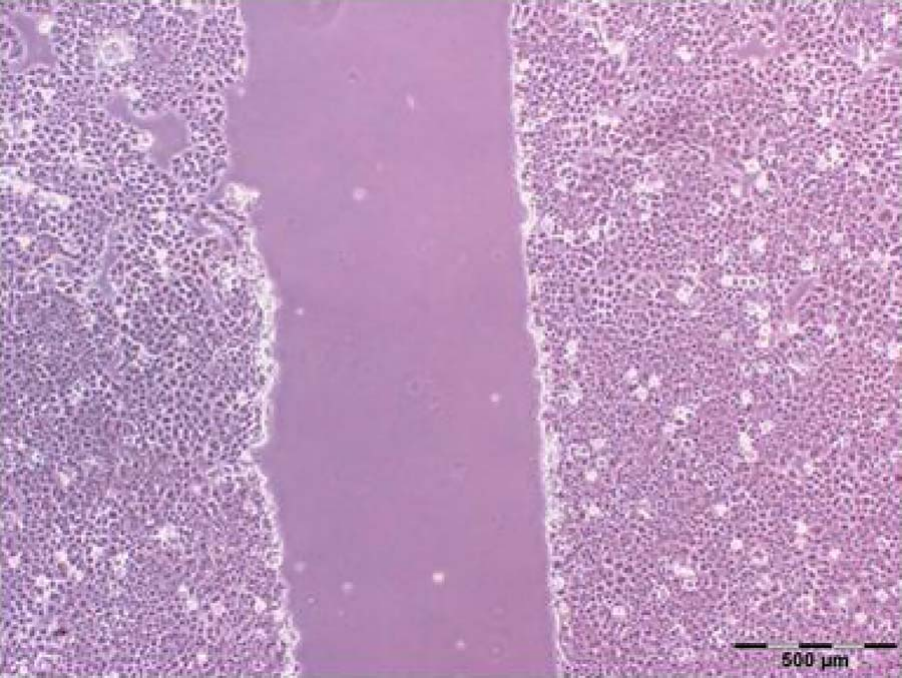
A scratched zone made in cellular mono layers of MCF7 cell line in culture, representing wound model in vitro. Micrograph was made by phase contrast microscopy.

**Fig. 7:**
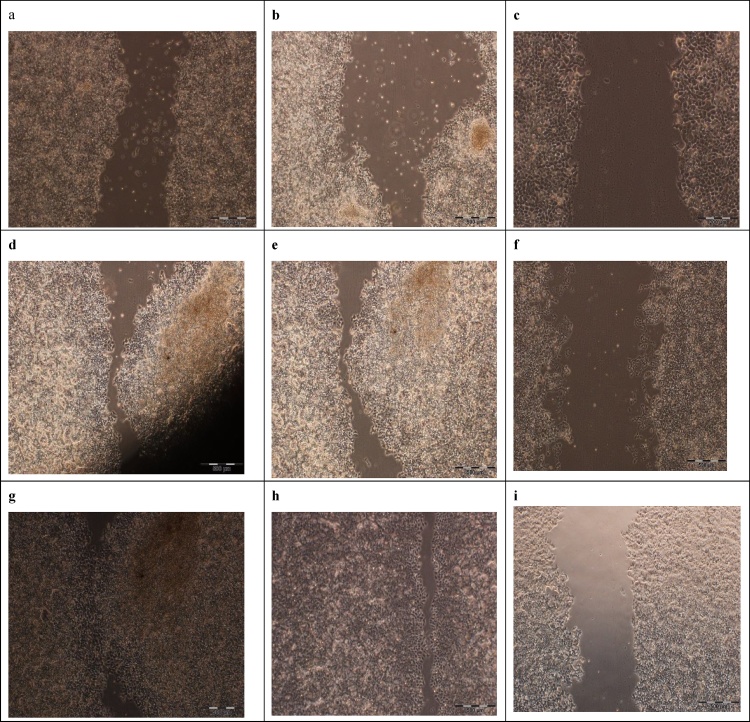
Photomicrograph for in vitro wound assay. MCF7 migration rates were assessed at a time period of 24 h after wound induction in different groups; medium culture as control (a), treatments with un infiltrated protein extract as negative control (b) at and treatments with infiltrated leaves extract (c) 48 h after wounding in different groups of HG medium (e), un infiltrated leaves (f) infiltrated leaves, also 72 h after wounding in different groups of: HG control (g), un infiltrated leaves (h) and infiltrated leaves(i).

Upon the creation of scratches, representing the wound models, in the cultured Cancer cells, their initial width were estimated between 700 and 800 μm (Fig. 6) The migration rate of the cells in the treated groups depended very much on the applied transformed tomato leaves. Upon creation the scratches, representing the wound models, in the cultured migration rate of MCF7cells in the three treated groups; first group was total extraction of infiltrated tomato leaves and second one was un-infiltrated tomato leaves as control and the other control was medium culture without plant protein extraction. Intermediate concentrations of the extract (2 mg/ml) generally stimulated the migration of cancer cell line into closure of the scratch, Percentage of closure calculated by Image J. software. 24 h post treatment the percentage of two control groups migration to scratch was 22% (Fig. 7a,b) and after 48 h, the scratch closure was recorded as 88% (Fig. 7e). Interestingly, 72 h following the same treatment, the scratch was almost completely closed, (Fig. 7g, h). However, 24 h after treatment with the same concentration of the protein extraction of infiltrated tomato leaves, no obvious migration of the cell line was observed (Fig. 7c) and in the same treatment, the scratch closure happened after 48 and 72 h by rates of 10%, and 24%, respectively (Fig. 7f and i). The results indicated that the extraction of plant recombinant protein was negatively correlated with the migration rate of the cells (Fig. 7).

## 4 Discussion

In recent decades, a large number of studies have been initiated to investigate the possibility to express recombinant protein in tomato as oral vaccine. The goal of this project was to explore the transient expression of CCL21 antigen in *S. lycopersicum* as oral vaccines although transient expression is not the preferred method for commercial production of recombinant vaccine in plants [3]. Moreover, this method makes it possible to evaluate efficacy of a potential recombinant vaccine in a short time [15]. In this study agroinfiltration is demonstrated to be fast and confident method to produce transgenic plant. In this study, CaMV 35S promoter, Kozak sequence, ER signal peptide were used and codons were optimized to enhance gene expression, similar to previous reports [16] Previous studies suggested that incorporating Kozak sequence in the upstream of the start codon can significantly increase the efficiency of translation in eukaryotic cells [12]. Therefore, a Kozak (GCAACA) plant translation initiation sequence was included before the start codon of *ccl21* to ensure high expression levels [[18], [19]]. Furthermore, several researchers included an ER retention signal, SEKDEL in the C-terminal as it is expected to sequester the protein in the ER to fold correctly, as well as to increase protein stability and prevent the entry of protein into the Golgi apparatus, the site for plant specific glycosylation [20], and C-terminal 6His tag to facilitate purification. PCR assay confirmed presence of the synthetic construct in infiltrated leaves but the gene of interest is not integrated in the nuclear genome of plant cell. Thus, the transgene in plant tissue is high and PCR product band in electrophoresis is almost as sharp as that of pure plasmid as control. The relative expression of *ccl21* in the infiltrated leaves was measured by qPCR assays. The highest gene expression of recombinant plant-produced *ccl21*, as measured by quantitative real-time PCR was detected 41.2 at 3 days post infiltration (dpi) RT- PCR assay was also use for evaluating the gene expression. As can be seen from dot blot assay, the protein sample obtained from transformed leaves generated a strong signal which is comparable to that of positive control, whereas wild type plant protein was not detectable. Most of the works in the field of transient antigen expression in plant hosts have been conducted by means of plant viruses as vehicle for gene delivery and expression, in which the epitope of interest is usually inserted within the coat protein gene [17]. Although a good level of transgene expression was achieved in our experiment, it should be mentioned that the results cannot be confidently attributed to the presence of these factors, since their effect on the foreign gene was not evaluated. In the present study, the ELISA results demonstrated that CCL21 expressed in *S.lycopersicume* is correctly translated, folded and fully functional. Therefore our results, including high level expression of biologically active CCl21 protein, suggest that plants may be an important complement to traditional vaccine production methods, and thereby making vaccine more accessible worldwide. Scratch assay was used to find the biological function of this protein [1]. CCl21 protein can be used for anti-metastatic of cancer cell lines [2]. In the present study, we aimed to investigate the potential activity of this extract in stimulating the proliferation and migration of cancer cell lines by wound healing method. Cancer cell lines migrate into the wound site 24 h after scratch seeding cells. In our proliferation experiments, after seeding cells in MCF7 growth medium (15% FBS) to ensure recovery from subculture or thawing, the growth media was replaced by medium without supplementation. In these conditions the protein extraction of infiltrated leaves at concentrations from 2 mg/ml, significantly prevent migration and growth of cancer cell lines in monolayer cell cultures, compared to that in control groups (basal medium and un infiltrated leaves). The results demonstrated decreased survival and anti-metastatic of cancer cells in the presence of CCL21 recombinant protein.

## 5 Conclusion

The present study is the first to report gene expression of *ccl21* construct in tomato via agro infiltration to use this plant as oral vaccine. Obtained results, including high level expression of biologically active CCL21, suggest that plants may be an important complement to traditional vaccine production methods, and thereby making vaccine more accessible worldwide. Also we report here using an in-vitro model of wound healing potential of CCl21 protein of tomato leaves. According to these results this construct was expressed in tomato and protein extracts seemed to be the most effective dose for stimulation of proliferation and migration of cancer cell lines.
